# Management of renal nutcracker syndrome by retroperitoneal laparoscopic nephrectomy with ex vivo autograft repair and autotransplantation: a case report and review of the literature

**DOI:** 10.1186/1752-1947-3-82

**Published:** 2009-10-27

**Authors:** Danfeng Xu, Yushan Liu, Yi Gao, Lei Zhang, Junkai Wang, Jiangping Che, Youhua Zhu

**Affiliations:** 1 Department of Urology, Changzheng Hospital, 415Rd, Fengyang, Shanghai, 200003, China; 2 Organ Transplantation Institute, Changzheng Hospital, the Second Military Medical University. 415Rd, Fengyang, Shanghai, 200003, China

## Abstract

**Introduction:**

Nutcracker syndrome (NCS) is caused by a compression of the left renal vein between the aorta and the superior mesenteric artery (SMA). It results in left renal venous hypertension, and the subsequent development of venous varicosities of the renal pelvis, ureter, and gonadal vein.

**Case presentation:**

A 21-year-old Chinese woman was admitted with a seven-month history of unilateral severe hematuria. On admission, she was identified as having nutcracker syndrome. The patient was treated with retroperitoneal laparoscopic donor nephrectomy and renal autotransplantation. The patient underwent retroperitoneal laparoscopic donor nephrectomy using a retroperitoneal three-port technique with ex vitro autograft repair and subsequent renal autotransplantation into the iliac fossa. In order to shorten the hot ischemia time and improve the patient's cosmetic outcome, a minor oblique incision in the left, lower quadrant was prepared in advance of the laparoscopic donor nephrectomy for use as a site for the autograft to be procured through the retroperitoneal space and as a transplant site for the autograft. Two days after the operation, the patient's symptoms subsided. Serum creatinine before and after the operation were 53 mmol/L and 55 mmol/L, respectively. The patient had normal renal function during a follow-up three months after the operation.

**Conclusion:**

The treatment of nutcracker syndrome by retroperitoneal laparoscopic nephrectomy with ex vitro repair and autotransplantation is a simpler and less invasive procedure than open surgery. Moreover, a minor incision on the left hypogastrium can shorten the autograft's hot ischemic time and improve patients' cosmetic outcomes, especially in young women.

## Introduction

Nutcracker syndrome (NCS) is caused by a compression of the left renal vein between the aorta and the superior mesenteric artery (SMA) [1]. This results in left renal venous hypertension and the subsequent development of venous varicosities of the renal pelvis, ureter, and gonadal vein. It is also called renal vein entrapment syndrome. The phenomenon was first described in 1950. De Schepper named this phenomenon 'Nutcracker Syndrome' in 1972 [2]. Normally, the left renal vein (LRV) passes anteriorly across the abdominal aorta. But, in rare cases, the left renal vein becomes located in a retro-aortic position, compressed between the aorta and the vertebral column. The condition that LRV is anterior to the abdominal aorta is called anterior nutcracker syndrome. The other is called posterior nutcracker syndrome [3].

The syndrome is manifested by unilateral hematuria, left flank abdominal pain, and occasionally a varicocele among men or abnormal menstruation among women. Left renal venous hypertension may cause multiple symptoms [4,5].

This syndrome can only be diagnosed after excluding all known causes of hematuria. A Doppler ultrasonic contrast [6], computed tomography (CT) or magnetic resonance angiography (MRA) [7] may be important for differential diagnoses.

Different treatments have been proposed for this syndrome [1]. Here, we report our experience treating a young woman with NCS through retroperitoneal laparoscopic nephrectomy and autotransplantation.

## Case presentation

**Figure 1:**
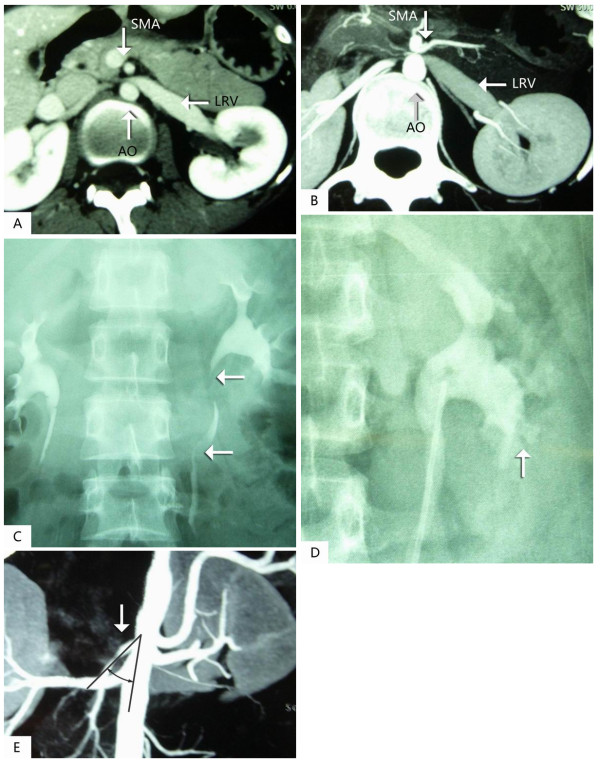
**A (pre-contrast) and B (post-contrast), Computer tomography (CT) angiography demonstrates compression of the left renal vein between the aorta (A) and superior mesenteric artery (S) with the dilation of the distal part of the left renal vein on axial cuts**. **C**, A retrograde pyelogram showed lobulated filling extravasate defects in calix at the lower pole of the left kidney. **D**, Excretory phase anterior volume rendered image shows tortuous, laterally displaced left proximal ureter (arrows) due to dilated varicose veins. **E**, A CT angiography demonstrates the acute angle between the aorta (A) and superior mesenteric artery (S) in this patient.

A 21-year-old Chinese woman was admitted with a seven-month history of unilateral severe hematuria. In March 2008, urine analysis revealed that the patient had asymptomatic microscopic hematuria. She was given oral antibiotics but, after one week of treatment, the patient's symptoms persisted. In a follow-up after four months, the patient still had hematuria. In July 2008, cystoscopy showed blood oozing from the left ureteric orifice. Real time ultrasonic imaging (duplex scanning) showed that the angle between the abdominal aorta and superior mesenteric artery was uncommonly sharp. The posterior wall of the superior mesenteric artery was very close to the anterior wall of the aorta. The inside diameter of the left renal vein, at the point where it crosses the aorta and SMA, was less than 2.0 mm. The highest peak velocity was detected at this point. The inside diameter of the left renal vein was 9.0 mm with lower peak velocity. The location of both kidneys at erect position was 30 mm less than that of the decubitus position. A CT was used for detecting the anatomical relation of the LRV with the aorta and SMA. The left renal vein was swelling and was compressed at the point where it crossed between the aorta and SMA, while the distal part of the point was strikingly expanded (Figure 1A and 1B). An IV urogram showed that the shape of both the renal pelvis and renal calyces were clearly visible. No pelvic or ureteral notching was identified. An excretory phase image showed that the left proximal ureter was laterally displaced (Figure 1C). A retrograde pyelogram showed lobulated filling extravasate in the calyx at the lower pole of the left kidney (Figure 1D). A CT angiography showed the acute angle between the aorta (A) and superior mesenteric artery (S) of this patient (Figure 1E).

The operation was performed with the patient under general anesthesia. The patient was placed in a prostrate position with an 8 cm, oblique incision in the left lower quadrant as the site for autograft. The autograft was taken out through a retroperitoneal tunnel after the laparoscopic nephrectomy was completed. Muscular layers of the incision were sutured and the wound was temporarily protected by membrane in order to maintain the normal pressure of CO_2 _pneumoperitoneum.

**Figure 2:**
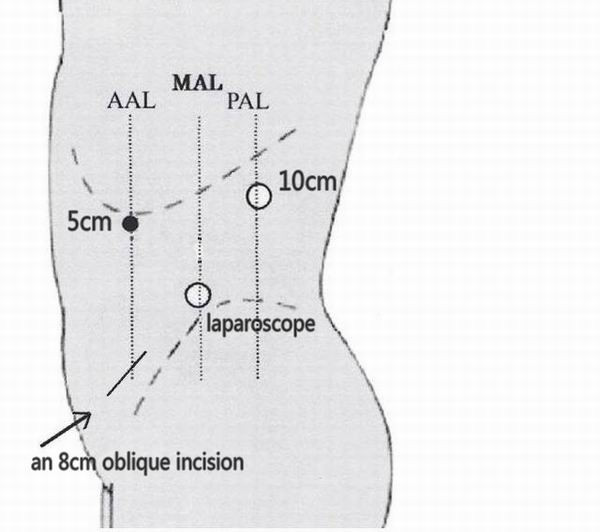
**The conceptual diagram of three working ports of the retroperitoneal laparoscopic nephrectomy**.

Then the patient was turned over and placed in a lateral decubitus position. A laparoscopic donor nephrectomy was conducted as described previously [8]. A 2 cm incision on the posterior axillary line across the twelfth rib costal margin in the left lumbar region was the first point. The peritoneum was pushed away by finger to expose the perirenal space. A balloon device for dilation of the retroperitoneum was inserted into the perirenal space, and 800 mL of air was pumped into the balloon to enlarge the perirenal space. After creating the pneumoperitoneum, a 25° laparoscope was inserted above the midaxillary line, across the spina iliaca. Additional working ports (5 mm) were placed under the anterior axillary line across the eleventh rib costal margin (Figure 2). The kidney was exposed by opening Gerota's fascia. The adrenal, lumbar, and gonadal branches in the left renal vein were clipped and transected. Along the inferior pole of the kidney, the left ureter was clipped and transected until left ureter was exposed over 12 cm long. The renal pedicle was dissected to expose the left renal artery and renal veins. Then, the renal artery was clipped by hem-o-lock and transected. A similar maneuver was performed with the renal vein, wholly freeing the kidney.

Donors received subcutaneous heparin after the operation. However, systemic anticoagulation was not employed before clamping the renal vessels. After the resected kidney was extracted through the oblique incision pre-prepared under the retroperitoneal tunnel, it was placed immediately on crushed ice and flushed with iced UW solution at 4°C. The kidney specimen was freed from all adjacent fatty tissues. Following reconstruction of the renal vessels, an autograft was transplanted retroperitoneally into the left iliac fossa. End-to-side anastomosis of the renal vein and the left external iliac vein using 5-0 polypropylene suture and end-to-end anastomosis of the renal artery and the divided left internal iliac artery using 6-0 polypropylene suture were completed, respectively. Ureteric anastomosis was carried out by formal antirefluxive implantation to the bladder as an extravesical onlay over a double J stent.

From incision to final wound closure, the overall time of the surgery was nearly 200 minutes. The retroperitoneal laparoscopic nephrectomy took almost 80 minutes. The renal autotransplantation took 60 minutes. The hot ischemia took 5 minutes, while the cold ischemia took 60 minutes. Total blood loss, as calculated from anesthesiology charts, was less than 100 ml.

Two days after the operation, the patient's symptoms subsided. Preoperative and postoperative serum creatinine was 53 mmol/L and 55 mmol/L, respectively. The average length of stay was eight days. Three months after the operation, an ultrasonic inspection and intravenous pyelography studies revealed that the autograft functioned normally, while a nephrogram indicated a normal infusion by level of a glomerular filtration rate (GFR) of autograft.

## Discussion

Different treatment options have been proposed for this syndrome, including follow-up, conservative treatment and surgical therapy [1,9,10]. The common viewpoint for management should be based on the age of the patient at the onset of the disease [11]. For patients under 18-years-old, follow-ups and conservative treatment are recommended until collateral circulation establishment or a superior mesenteric artery augmentation is achieved to palliate the compression of the left renal vein between the AO and SMA. If for two years after the conservative treatment, the symptoms persist or complications such as anemia, loin pain, varicocele and functional lesion in the renal vein appear, surgical procedures are reasonable. However, for adults 18-years-old and above, only after a six-month medical treatment and after nutcracker syndrome is diagnosed can surgical procedures be recommended.

The available surgical procedures include intra- or extravascular stents and open surgical procedures. Expandable metallic stents were first reported by Neste et al. Now, a variety of stents can be deployed in the narrow portion of the left renal vein, such as intravascular stenting treatment for nutcracker syndrome [12-14]. Intravascular stenting is a simple, micro-invasive, repetition management procedure. Its shortcomings include venous occlusion caused by fibromuscular hyperplasia, proximal migration or embolization of the stents, and the need for anticoagulation medication [15]. Extravascular stenting using a ring-reinforced Polytetrafiuoroethylene (PTFE) graft by open or laparoscopic surgery was tested in sporadic cases [12].

Open surgical procedures employed to rectify the problem include the transposition of left renal vein [16], transposition of the superior mesenteric artery [17], renal autotransplantation [18] and gonadocaval bypass [19]. The transposition of the left renal vein is an efficient and less complex surgical approach to treat anterior nutcracker syndrome. Left renal vein transposition involves dividing the left renal vein at its junction with the inferior vena cava. It also involves the repair of the vena cava defect and the re-anastomosis of the left renal vein to the inferior vena cava at a lower level away from the superior mesenteric artery. A transposition of the superior mesenteric artery is based on similar surgical principles, but is more difficulty compared to a left renal vein transposition. Vessel transpositions involve risks that include bleeding, thrombosis and a paralytic ileus [20]. A thrombosis of the superior mesenteric arterial would be disastrous for patients undergoing this operation.

Renal autotransplantation has been used in the management of renal vessel trauma, thrombosis, stenosis, and aneurysms. It has also been advocated for ureteral avulsion, urothelial malignancy, renal calculus disease, renal tumor, renal trauma, retroperitoneal fibrosis, and nutcracker syndrome [21]. Recent developments in micro-invasive surgery have made laparoscopic donor nephrectomy a more attractive technique option [22]. Currently, despite the increasing number of case reports describing such scenarios, the repair of nutcracker syndrome in patients through laparoscopic donor nephrectomy and autotransplantation is not well-described in the literature.

Laparoscopic donor nephrectomy was first carried out in 1995. In less than a decade, most centers adopted laparoscopic surgery. The conventional open approach to autotransplantation requires either a large, single, midline incision from the xiphoid process to the pubic symphysis or two separate incisions: one flank incision to procure the kidney, and another in the iliac fossa for transplantation. There is no doubt that this represents a considerable disincentive to potential donors. Compared to the open approach [23,24], laparoscopic nephrectomy and autotransplantation are feasible and minimally invasive alternatives. This approach avoids the need for an extended abdominal or flank incision, resulting in less postoperative morbidity without compromising the outcome. A laparoscopic nephrectomy is also associated with improved cosmesis, less postoperative discomfort, a shorter hospital stay, and decreased convalescence.

In our case a woman with nutcracker syndrome was treated with a retroperitoneal laparoscopic nephrectomy with ex vitro autograft repair and autotransplantation. Compared with a transperitoneal laparoscopic nephrectomy and autotransplantation, our approach had only one minor oblique incision in the iliac fossa both for the autograft to be procured through and for transplantation. Moreover, using only a small incision on the left hypogastrium can improve the cosmetic outcome of patients and may be well-accepted by patients, especially young women. In order to shorten the hot ischemic time of the autograft, the oblique incision had to be prepared in advance of the laparoscopic nephrectomy. Operation in a restricted retroperitoneal working space led to a shorter operation time and a lower chance of lesions in intraperitoneal organs. With no need of blood vessel manipulation, there was no risk of thrombosis. Postoperative anticoagulation medication was not needed and the cost of the surgery was reduced. This approach resulted in a faster recovery, less fatigue, and better quality of life for the donor.

With no accepted protocol for nutcracker syndrome repair, retroperitoneal laparoscopic donor nephrectomy and renal autotransplantation should be determined on a case-by-case basis. Based on the advantages of retroperitoneal laparoscopic surgery, this approach should become a standard method for nutcracker syndrome repair. From our experience, during renal allotransplantation [25], care must be taken to avoid irreversible ischemic injury of the patient's kidney autograft to prevent long-term graft damage.

## Conclusion

Treatment of nutcracker syndrome through retroperitoneal laparoscopic nephrectomy with ex vitro repair and autotransplantation is a simpler and more micro-invasive procedure than open surgery. Moreover, a minor incision on the left hypogastrium can shorten the hot ischemic time of the autograft and improve the patient's cosmetic outcome, especially young women.

## Abbreviations

NCS: Nutcracker syndrome; AO: the aorta; SMA: superior mesenteric artery; LRV: left renal vein; MRA: magnetic resonance angiography; IV: intravenous; GFR: glomerular filtration rate; PTFE: Polytetrafiuoroethylene; CT: computer tomography.

## Competing interests

The authors declare that they have no competing interests.

## Consent

Written informed consent was obtained from the parents of the patient for the publication of this case report and any accompanying images. A copy of the written consent is available for review by the Editor-in-Chief of this journal.

## Authors' contributions

DX, YL, GY and LZ managed the patient and reviewed the literature. JC and JW were the main writers of the manuscript. YZ moderated the manuscript. All authors read and approved the final manuscript.
